# Metal-Doped Graphitic Carbon Nitride Nanomaterials for Photocatalytic Environmental Applications—A Review

**DOI:** 10.3390/nano12101754

**Published:** 2022-05-21

**Authors:** Geetha Palani, Retna Apsari, Marlia M. Hanafiah, Katta Venkateswarlu, Sivarama Krishna Lakkaboyana, Karthik Kannan, Anilkumar Thaghalli Shivanna, Abubakr M. Idris, Chappidi Hazarathaiah Yadav

**Affiliations:** 1 Department of Physics, Faculty of Science and Technology, J.N.N. Institute of Engineering, Kannigaipair 601102, India; kesangee@gmail.com; 2 Department of Engineering, Faculty of Advanced Technology and Multidiscipline, Universitas Airlangga, Surabaya 60115, Indonesia; retna-a@fst.unair.ac.id; 3 Department of Physics, Faculty of Science and Technology, Universitas Airlangga, Surabaya 60115, Indonesia; 4 Department of Earth Sciences and Environment, Faculty of Science and Technology, Universiti Kebangsaan Malaysia, Bangi 43600, Malaysia; mhmarlia@ukm.edu.my; 5 Centre for Tropical Climate Change System, Institute of Climate Change, Universiti Kebangsaan Malaysia, Bangi 43600, Malaysia; 6 Laboratory for Synthetic & Natural Products Chemistry, Department of Chemistry, Yogi Vemana University, Kadapa 516005, India; 7 Department of Chemistry, Vel Tech Rangarajan Dr. Sagunthala R&D Institute of Science and Technology Avadi, Chennai 600062, India; drchryadav@gmail.com; 8 School of Advanced Materials Science and Engineering, Kumoh National Institute of Technology (KIT), 61 Daehak-ro, Gum-si 39177, Korea; karthikkannanphotochem@gmail.com; 9 Department of Chemical and Materials Engineering, Chang Gung University, Kwei-San, Taoyuan 33302, Taiwan; anil24march@gmail.com; 10 Department of Department of Chemistry, College of Science, King Khalid University, Abha 61431, Saudi Arabia; abubakridris@hotmail.com

**Keywords:** photocatalysts, graphitic carbon nitride, nanomaterials, metal-doped materials, environmental applications

## Abstract

In the current world situation, population and industrial growth have become major problems for energy and environmental concerns. Extremely noxious pollutants such as heavy metal ions, dyes, antibiotics, phenols, and pesticides in water are the main causes behind deprived water quality leading to inadequate access to clean water. In this connection, graphite carbon nitride (GCN or g-C_3_N_4_) a nonmetallic polymeric material has been utilized extensively as a visible-light-responsive photocatalyst for a variety of environmental applications. This review focuses on recent developments in the design and photocatalytic applications of metal-doped GCN-based nanomaterials in CO_2_ photoreduction, water splitting toward hydrogen production, bacterial disinfection, and organic pollutant degradation. Additionally, this review discusses various methods of using GCN-based materials to optimize dye sensitization, metal deposition, ion doping, and their environmental applications.

## 1. Introduction

In attending to the needs of the rising population of the world, rapid industrialization is taking place that is leading to the environmental pollution [1,2,3,4]. One of the major sources of pollution is wastewater released mainly from chemical industries; this industrial wastewater contains high concentrations of organic waste and heavy metal ions; by their nature, these are highly poisonous and carcinogenic, causing negative impact on sustainability of water resources [5,6,7]. Recently, environmental remediation technologies such as adsorption, chemical oxidation, incineration, and biological oxidation have been used in all types of organic and toxic wastewater treatment and also have various advantages in solar energy utilization, sensing, environmental treatment, and biomedical applications. The treatment of wastewater containing the excessively disposed heavy metals and toxic organic pollutants from industries is a major challenge in the current state of the world [8,9,10]. Recently, photodegradation of organic pollutants using a variety of semiconducting materials as catalysts has progressed with high prominence for the eradication of pollutants from wastewater [11]. However, graphitic carbon nitride (GCN), a nonmetallic semiconductor having a bandgap of ~2.7 eV that can absorb blue light and high thermal and chemical stability, has provided an interest in photocatalytic applications [12]. Further, GCN is an N-richer precursor that was the first synthesized polymer through heating of chemical substances such as thiourea, urea, cyanamide, dicyandiamide, and melamine [12,13].

During the last decade, extensive study has been conducted on the creation of realistic strategies to address the energy problem and to achieve environmental restoration. High energy needs, fossil fuel depletion, and environmental degradation have all emerged as serious worldwide issues in recent years. The utilization of clean, unlimited solar energy is critical in avoiding consequences of greenhouse gases (GHGs) and ensuring future energy supplies. Every year, hundreds of scientific journals publish articles on solar energy conversion technologies, such as the photocatalytic breakdown of organic pollutants, photocatalytic water-splitting hydrogen (H_2_) generation, photovoltaic cells, and dye-sensitized solar cells (DSSC) [12,14]. One of the most noteworthy applications was developed by Fujishima and Honda in 1972, focused toward photoelectrochemical water splitting [15,16]. Since then, water splitting toward producing hydrogen has emerged as the most replaceable renewable energy technology with less environmental damage. GCN, similar to many other semiconducting materials, has low photocatalytic properties leading to inadequate light absorption and a significant risk of photogenerated electron and hole pair recombination. Generally, the cocatalyst has been widely examined to generate the composites with GCN for oxidation and reduction of active sites, trapping charged carriers, and inhibiting photogenerated electron-hole pair recombination [17].

Elemental doping can significantly alter GCN’s bandgap structure. This increases the hole and electron separation caused by the light. Wang and coworkers observed that the F-doping to the GCN matrix results in C-F bond formation, which can boost the light absorption range [18]. In another study, metals such as Fe were loaded onto GCN (Fe-GCN) and used the π-π structure to use the Fenton-like reaction. This suggests that GCN has excellent catalytic efficiency in the realm of cleaning. Here, Fe-GCN was synthesized to investigate the efficiency of challenging wastewater treatment using a heterogeneous photocatalysis–Fenton system. It also helps to separate photoelectrons from holes. In complex wastewater, the product delivered efficient organic matter removal [19,20]. In these cases, the s-triazine (C_3_N_3_) rings of GCN were certified as being the most stable in the environment with typical tri-s-triazine (C_6_N_7_) units.

**Figure 1:**
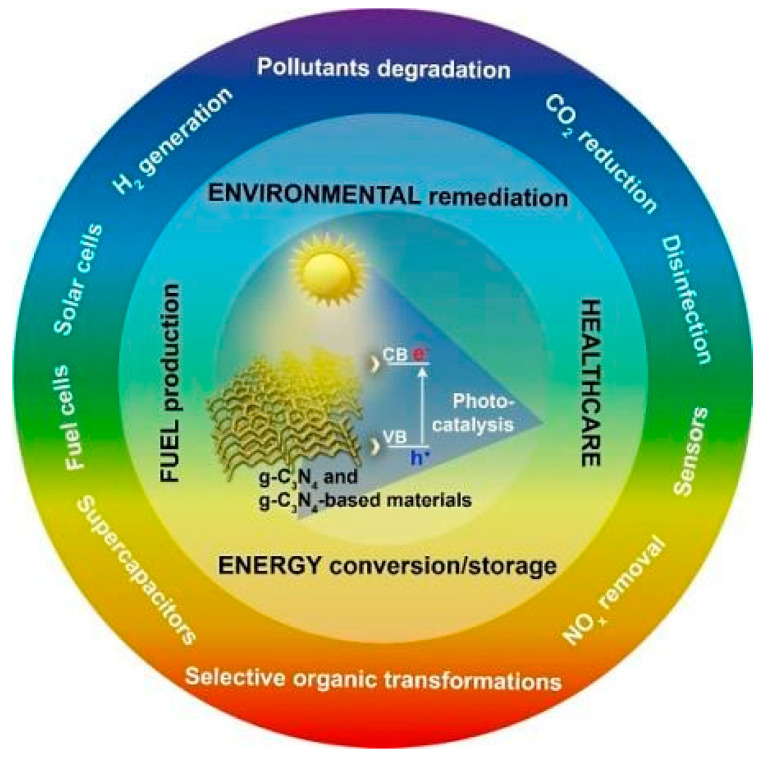
Considerations for GCN-based photocatalysts in various applications. Adapted from [23]. 2021, MDPI.

Various studies of GCN-based photocatalysts dealing the modification and synthesis, and their uses in environmental and energy challenges, are currently available and only a few publications have emphasized mostly on wide-ranging characteristics and g-GCN-based photocatalyst performance enhancement [21,22]. In the end, it becomes appropriate to provide a rather complete and fully updated evaluation of the most recent achievements in metal-doped GCN-based photocatalysts for heterogeneous photocatalysis. Potential applications of GCN photocatalysts are briefly discussed in this review paper. This review also provides the new GCN-based architectures and materials for further developments with better use of photocatalytic efficiency and solar energy, and it helps to show the challenges for the worldwide GCN-based photocatalysts utilization in the storage and production of sustainable energy from renewable resources. This review also provides a few new ideas concerning architectures in developing new materials for the fuel cells, emitting devices, solar cells, sensing devices, and batteries for different sustainable-energy-associated fields. Figure 1 depicts design criteria for GCN-based photocatalysts focusing on various photocatalytic phases.

### General Facts/Features of GCN

In the 1830s, GCN was first found by Berzelius and recognized by Liebig as one of the earliest synthetic polymers [24]. Because of its unknown chemical structure, insolubility, and chemical inertness, it was not widely employed until now. Wang et al.’s theoretical predictions highlighted the properties of GCN and GCN uses for photocatalysis [25], which is a pioneering work on photocatalytic H_2_ evolution. The framework structure, similar to graphene, is composed of N-bridged poly aromatic heptazine π-conjugated defect-rich units that create sheets with very strong covalent C-N connections. Additionally, its properties include the ease of changing the bandgap, cost-effective synthesis, nontoxicity, absorption of near-visible light, thermal and chemical stability, and high excitation recombination rates. GCN’s practical application/actual use, however, is limited due to detriments such as low specific surface area, surface inertness, weak charge transport and kinetics, and high excitation recombination levels. To overcome these flaws, doping to strengthen the heterostructure production are commonly used to improve the electronic structure, exfoliation to increase surface area, porous architecture construction to promote surface characteristics, enhance charge transport kinetics, etc.

## 2. The Catalytic Mechanism of Photocatalysts Based on GCN Heterogeneous

**Figure 2:**
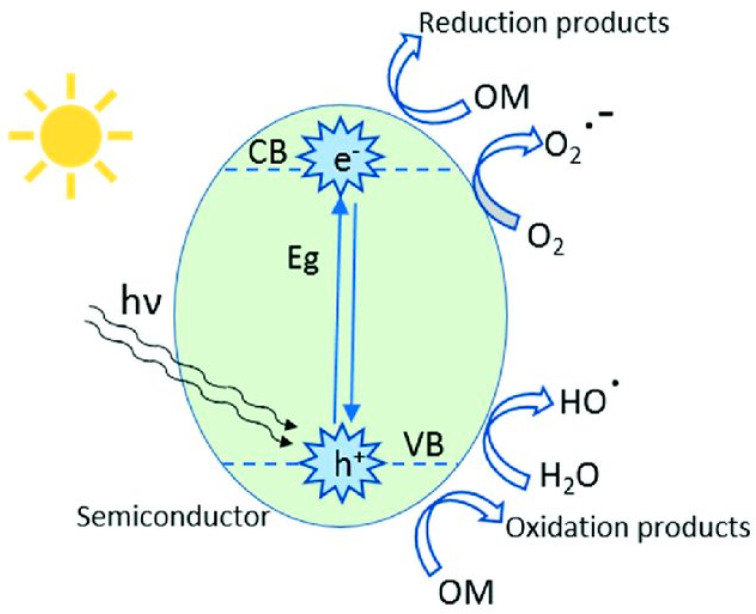
Schematic representation of heterogeneous photocatalysis mechanism. Adapted from Ref. [27]. 2021, MDPI.

Most GCN research has focused on narrowing the energy bandgap while simultaneously decreasing charge photogenerated electron-hole pair’s recombination: (i) doping metallic elements into GCN as electron entrapment centers, generating highly oxidized holes, (ii) creating heterojunctions, such as metal–semiconductor, semiconductor–semiconductor to efficiently divide holes and electrons [26]. Recent research has shown that incorporating the GCN metal ions into the polymerization network enhances lifespan and charge carrier mobility while also lowering the material bandgap. Tri-s-triazine moieties coupled by three-fold nitrogen bridges provide a wide area in the GCN network having six lone pairs of electrons on nitrogen atoms, and these can help in optimal coordination for transition metal ion accommodation. Figure 2 depicts the schematic diagram of GCN photocatalytic performance.

**Figure 3:**
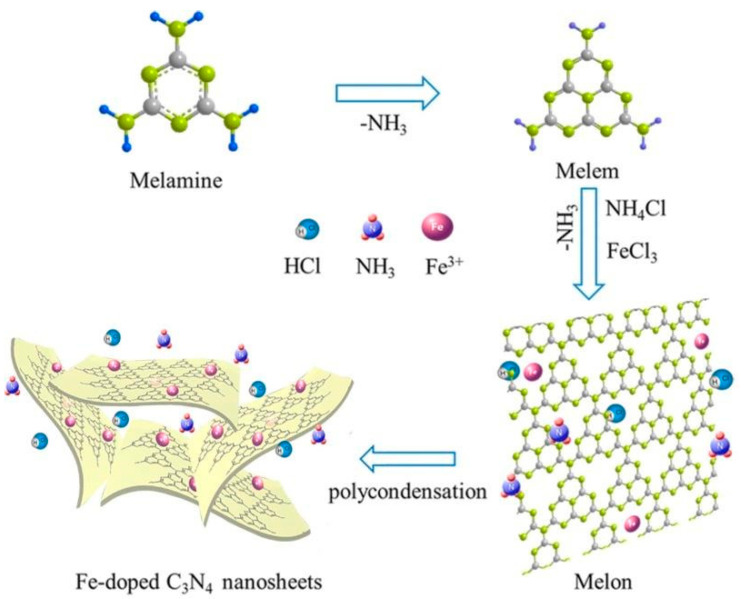
Fabrication of Fe-doped GCN. Adapted with permission from [28]. 2017, John Wiley & Sons.

**Figure 4:**
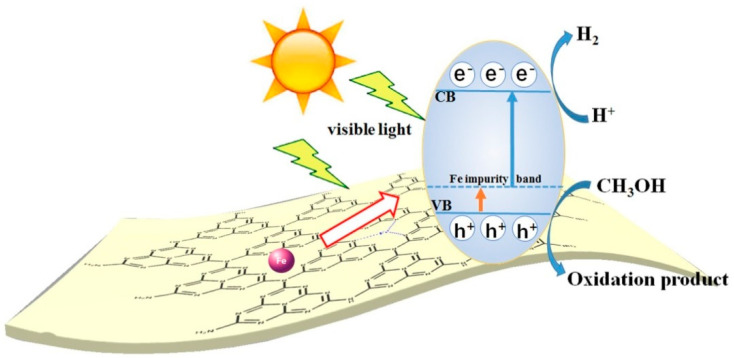
The charge mobility in Fe-doped GCN photocatalytic performance. Adapted with permission from [28]. 2017, John Wiley & Sons.

Most GCN research has focused on narrowing the energy bandgap while simultaneously decreasing charge photogenerated electron-hole pair’s recombination: (i) doping metallic elements into GCN as electron entrapment centers, generating highly oxidized holes (ii) creating heterojunctions, like metal-semiconductor, semiconductor-semiconductor to efficiently divide holes and electrons [26]. Recent research has shown that incorporating the GCN metal ions into the polymerization network enhances lifespan and charge carrier mobility while also lowering the material bandgap. Tri-s-triazine moieties coupled by three-fold nitrogen bridges provide a wide area in the GCN network having six lone pairs of electrons on nitrogen atoms and these can help in optimal coordination for transition metal ion accommodation. Figure 3 and Figure 4 show the fabrication of Fe-doped GCN (Figure 3) and its critical role in photocatalytic methanol oxidation (Figure 4).

Since Wang et al. (2009) discovered a change in the GCN functionality when doped with certain metals, including Fe [29], Fe-doped GCN nanosheets have been explored as a means to develop extremely efficient photocatalysts and to investigate the mechanism that increases their photocatalytic activity. Tonda et al. (2014) [30] investigated Fe-doped GCN nanosheets produced from melamine and FeCl_3_ using a two-step process. The involvement of Fe^3+^ as a photogenerated electron trap explains the large increase in photocatalytic performance for RbH degradation. Recently, Ma et al. [31] published an article in 2019 on the Fe-doped GCN photocatalyst produced by single-step melamine thermal condensation and iron nitrate nanohydrate, emphasizing the importance of the Fe^3+^/Fe^2+^ pair in the photocatalytic activity. Interestingly, research on Fe^−^ and P-codoped GCN revealed a considerable increase in photocatalytic efficiency. Although increased photocatalytic efficiency and mechanism in Fe-doped GCN material are still being verified, it has made a significant contribution to the detection of photocatalysts in general and solar energy conversion materials in particular [32].

### 2.1. Benefits and Drawbacks of g-GCN-Based Photocatalysts

Based on the study of bandgap and nanostructures, both are very important for photocatalytic applications. Figure 4 depicts the GCN bandgap structures along with various standard potentials of common redox processes at pH 7. From Figure 4, it can be seen that GCN has a modest bandgap of 2.7 eV, which corresponds to 460 nm optical wavelength, making it visible-light active. The potential and thermodynamic losses are considered in the photocatalytic process, and the 2.7 eV bandgaps fall, allowing for both visible-light absorption and water splitting. From the Figure 4, it is noted that the top CB level of GCN is more negative than those potentials and conventional inorganic semiconductor of CO_2_-reduction, oxygen reduction reactions, and hydrogen evolution, which suggests GCN possesses photogenerated electrons with a high thermodynamic driving force that can reduce different types of small substances, such as O_2_, H_2_O, and CO_2_ [33]. As a result, the GCN band structure makes it suited to a wide variety of applications, including reduction of carbon, photocatalytic water splitting, disinfection, and organic synthesis.

GCN-based photocatalytic efficiency is predominantly determined by crystal structure, electrochemical and photoelectrochemical stability, optical and surface physicochemical adsorption, and electronic characteristics of GCN. As a result, a basic understanding and control of these structural and chemical factors allows for scalable GCN-based composite photocatalyst production. This includes better photocatalytic behavior, which is useful for developing some robust GCN-based material systems for functional photocatalytic implementation and fundamental understanding of photocatalytic enhancement mechanisms at the single-atom level [34].

### 2.2. Photocatalytic Hydrogen Gas (H_2_)

H_2_ gas production has recently attracted widespread research interest. Although fossil fuel energy has greater heat energy production, fossil fuels are being depleted. Because of its clean reactions and simplicity, conventional solar energy remains the most promising technology for the mechanisms of water splitting to generate hydrogen. She and colleagues [35] described the 2D-Fe_2_O_3_/GCN Z-scheme catalyst synthesis. From the literature, their report shows hydrogen evolution activity was greatly increased in hybrids with Fe_2_O_3_ nanostructures, which can reach 31,400 mol g^−1^ h^−1^ for Fe_2_O_3_/2D GCN (Fe_2_O_3_-loading 3.8 wt%). According to these studies, heterostructure carbon nitride semiconductors for hydrogen production exhibit high photocatalytic efficiency [36]. Table 1 summarizes the various g-GCN heterojunction photocatalytic hydrogen production investigations. The difficulty of splitting oxygen-containing products and hydrogen (hydrogen storage mechanism) in photocatalytic hydrogen (H_2_) synthesis is impeded by the proximity to reduction–oxidation sites. As a result, it is more difficult to obtain photogenerated electrons and holes at the oxidation and reduction sites in the proposed photocatalyst, which could result in oxygen- and hydrogen-containing products reversing reactions or even causing explosive damage. To overcome this obstacle, researchers have searched for separately produced H_2_ from oxygen products while keeping the tight spacing between oxidation and reduction sites, which is critical for photoinduced charge transfer. Graphene sandwich devices with photocatalytic hydrogen production and storage capabilities were created by Li Yang and colleagues in 2017 [37].

This is performed not just to avoid the backward reaction, but also to make it possible for the storage of created H_2_ on the surface, with a 5.2 wt% storage rate, which is very near to that of the U.S. Department of Energy’s guidelines (6.5 percent by weight). Xijun Wang’s group also used first principle calculations to manufacture a carbon–quantum-dot/GCN hybrid with a strong capability to isolate H_2_ from the oxygen in photocatalytic water splitting. The protons were only allowed to penetrate the graphene inner layer to create H_2_ in their work [39]. The H_2_ gas created was then encapsulated in the photocatalyst’s inner layer; this also prevented the process from reversing and increased the H_2_ availability that was generated.

### 2.3. CO_2_ Reduction

The ecosystem, notably the atmosphere, has suffered as a result of population increase and industrialization. CO_2_ emissions have recently remained the most pressing issue in the cosmos. CO_2_ produced by fuel combustion at all levels, from home to industry, has significantly facilitated the rise of atmospheric air pollution, which is largely responsible for the current global warming crisis [40]. To reduce CO_2_ emissions, various strategies have been devised. SDG 7 calls for the use of clean, renewable energy by lowering CO_2_ emissions in the atmosphere. However, rising fuel use and industrial production continue to contribute significantly to CO_2_ emissions (Table 2). Technologies to degrade the CO_2_ produced have been developed. Photocatalytic reactions, for example, have been shown to be one of the most promising technologies for CO_2_ removal. Sheng Zhou and colleagues [41] described a simple in situ fabricated GCN-N-TiO_2_ heterojunction as an efficient photocatalyst for CO_2_ reduction. Thermal treatment of well-mixed Ti (OH)_4_ and urea with varying mass ratios in an alumina crucible resulted in composites of GCN and N-doped TiO_2_ (such as GCN-N-TiO_2_). To obtain the product, this mixture was heated at 550 °C for 3 h, followed by heating at 580 °C for 3 h at 5 °C per min rate. To remove traces of adsorbed alkaline and sulfate species, the product was washed several times with 0.1 M HNO_3_ followed by distilled water. Finally, the material obtained was dried overnight at 80 °C. The photocatalytic CO_2_ reduction property of the fabricated material was investigated in a closed gas-circulation system using water vapor and CO_2_ under simulated light irradiation. Because it facilitates the parting of electrons that are induced by light from holes, the heterojunction between GCN and nitrogen-doped TiO_2_ displayed high activity.

These findings show that when compared to its precursors, the hereto structured GCN semiconductor has a high photocatalytic performance in CO_2_ reduction. More investigations on heterojunction for CO_2_ photocatalytic reduction are presented in Table 2.

## 3. Dye Sensitization

Because of its chemical stability, photophysical properties, and high molar extinction coefficient, GCN plays an important role in dye sensitization systems, serving as a light-harvesting center for extending the response to light of longer wavelengths and serving in electron injection to semiconductors from the excited dye substance. There are two types of dye-sensitized GCN composite fabrication methods: physical impregnation with weak contacts and chemical impregnation with covalent linkage. Many dyes, such as porphyrins, rhodamine B [45], erythrosin B, and eosin Y, have been reported to be employed in GCN sensitization.

Qian and coworkers [46] investigated fluorescein-sensitized Au/GCN nanocomposite material for improved photocatalytic H_2_ generation, finding that the addition of fluorescein to the photocatalytic system raised the H_2_ synthesis rate by greater than 22 fold. The considerably increased performance was attributed to effective photoexcited electron transfer between photoexcited fluorescein molecules, GCN, and Au, as evidenced by transient photocurrent responses and photoluminescence spectra. Dang and coworkers built EY-sensitized Au/GCN NSs to use as photocatalysts for water (solar) splitting, implementing a simple one-step calcination process. The electrons of the photocatalytic process were transported to GCN CB from the excited EY substance and then to the Au cocatalyst, significantly increasing photocatalytic H_2_ production activity and effectively suppressing charge carrier recombination. The significant advantage of the dye-sensitized photocatalytic system over a photocatalyst system having only semiconductors is the wide light-absorption range. The use of dye sensitization on GCN proved to be a successful method for harvesting solar energy of longer wavelengths. GCN’s photocatalytic activity was effectively boosted by electron injections from light absorption and dye excitations. Dye sensitization could also improve photoexcited hole and electron transit and separation between GCN and dyes [47].

## 4. Photocatalytic Activity Analysis

**Figure 5:**
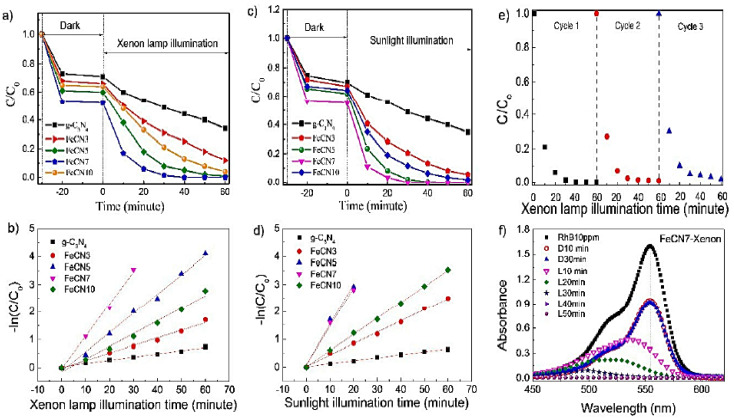
Photocatalytic performance and rate of reaction of Fe-doped GCN nanosheets using different concentrations of Fe in RhB decomposing solution under (**a**,**b**) xenon lamp irradiation and (**c**,**d**) sunlight condition; (**e**) the Fe-doped GCN’s reusability performance, and (**f**) change in absorption intensity and absorption maxima of the RhB solutions in different times, adapted from [49]. 2020, MDPI.

Figure 5 shows the photocatalytic application of Fe-doped GCN for RhB degradation using xenon lamp irradiations (luminance Ev2500 lx). For all samples, the results show that adsorption–desorption equilibrium may be attained in <10 min of stirring in the dark. FeCN_7_ has the best adsorption capability after 30 min of stirring in the dark, reducing RhB content by 43%. The FeCN_7_’s largest particular surface area, shown by the BET results, can explain this fairly well [48]. The RhB degradation using xenon light is only 60% after 60 min of xenon lamp irradiation when pure GCN photocatalyst is present; however, the photocatalytic property of Fe-doped GCN is significantly increased. The degradation of RhB using Fe-doped GCN photocatalyst enhances gradually by increasing the Fe-doping level, reaches its peak for FeCN_7_, and subsequently drops as the Fe amount increases. After 30 min of xenon light exposure, almost all of the RhB in the FeCN_7_ sample has been destroyed. The photocatalytic reaction rate is calculated using the pseudo-first-order kinetic model, ln(Co/C) = kt, where the k (rate constant) is calculated using the slope of the linear relationship of ln(Co/C) with reaction time (Figure 5b).

The FeCN_7_ has the highest k (k0.117) value, which is around 10-fold to that of GCN (k0.012). Figure 5c,d shows the photocatalytic behavior of Fe-doped GCN nanosheets under sunshine illumination to highlight the capacity to employ sunlight during photocatalytic processes. This also demonstrates that the FeCN_7_ sample has the best photocatalytic property, with a reaction rate of k0.140, which is thirteen times larger than that of pure GCN nanosheets. This shows that GCN-modified photocatalysts could be used in green technology to use and convert solar energy into various forms.

The recycling test for the FeCN_7_ sample was carried out under xenon lamp illumination to ensure the reusability of the Fe-doped GCN as-prepared photocatalyst, as shown in Figure 5e. The percentage of photodegradation of RhB is >95 in three cycles, indicating that as-synthesized Fe-doped maintained photocatalytic capability well or maintains good stability. Figure 5f depicts the temporal evolution of the RhB absorption spectrum for FeCN_7_ under xenon lamp irradiation [50]. Aside from the considerable decrease in RhB absorbance over time, a blue shift in the absorption maximum as lit by a xenon lamp was also noticed, revealing RhBdeethylation on the photocatalyst surface. The absorption maxima of intermediatory products of the RhBdeethylation phenomenon–RhB (554 nm); N, N, N^0^-Triethyl-rhodamine (539 nm); N, N^0^-diethyl-rhodamine (522 nm); N-ethyl-rhodamine (510 nm); and, lastly, rhodamine (510 nm)—are known to cause the blue shift (497 nm). The blue shift, together with a significant fall in absorbance maxima, suggests that two photodegradation processes for RhB coexist and compete in this case: (i) cleavage of entire conjugate chromophore structures, and (ii) N-demethylation. Because the HOMO (highest occupied molecular orbital) is found on N and LUMO (lowest unoccupied molecular orbital) is found on C and N atoms, photogenerated electrons in the GCN crystal lattice find it difficult to move among different heptazine units, resulting in a high recombination rate of electron-hole pairs and low photocatalytic efficiency. Fe^3+^ electron-trapped centers were produced by doping Fe into the crystal lattice of GCN in an interstitial configuration, which plays a significant role in photocatalytic activity [51]. 

As a result, the contribution of electron transport activities to boosting RhB breakdown can be explained as follows: (i) A photon absorbs the electron of the VB, causing its transfer to the CB, where it becomes a free electron, generating a hole with positive charge in VB. Both electrons and holes are free-charge carriers that can participate in a redox process to breakdown an organic compound. (ii) The photogenerated electrons from C and N atoms are trapped by the highly oxidized Fe^3+^ ion, resulting in a longer lifespan of positive charge holes capable of oxidizing OH^•^ to OH^•−^. (iii) After this, an electron migrates from Fe^3+^ to Fe^2+^, which can decrease the O_2_ to O_2_^•^ and later return to Fe^3+^. As a result, the rate of recombination of photogenerated electron and hole pairs is low, as evidenced by PL data. (iv) Electrons in Fe^2+^ energy levels absorb photons in the visual range and transfer to the conduction band, reducing O_2_ to O_2_^•−^ and generating a highly oxidized Fe^3+^ ion. It is visible in absorption spectra, which show a progressive increase in absorbance at 430–600 nm as Fe content rises [52]. The increase in photocatalytic performance is also corroborated by BET measurements, which show that FeCN_7_ photocatalyst has a large pore volume and specific surface area.

### 4.1. Photocatalytic Water Splitting

Several heterogeneous photocatalysts have been widely employed in attracting photocatalytic hydrogen generation from water reduction since Honda and Fujishima’s pioneering study in 1972 [53]. Half-reaction water splitting (for H_2_ and O_2_) and whole water-splitting systems are two types of photocatalytic water-splitting systems. Photocatalyst development with higher efficiencies from water and solar energy for hydrogen synthesis remains a major issue, and it is sometimes referred to as the "holy grail" of photocatalysis. Almost all oxide photocatalysts that can produce water splitting are only active when exposed to UV light. However, the ultraviolet part of the solar spectrum contains just a small percentage of incoming solar energy (about 4%). As a result, robust and highly efficient visible-light-driven photocatalysts are required for the practical, large-scale generation of hydrogen utilizing solar energy [54]. To split water, the photocatalyst’s bandgap energy (Eg) must be greater than 1.23 eV. On the other hand, visible light must be 3.0 eV (>400 nm) to be used. The bottom level of the conduction band (CB) must be more negative than the reduction potential of H^+^/H_2_ (0 V vs. normal hydrogen electrode (NHE) for pH = 0) for thermodynamic reasons, whereas the top level of the valence band (VB) must be more positive than the oxidation potential of O_2_/H_2_O for thermodynamic reasons (1.23 V). When considering its electronic properties, chemical stability, thermal, and visible-light-driven properties, GCN can be considered an excellent photocatalyst for enhancing the photocatalytic H_2_− production activity by hampering the backward reaction, accelerating photoinduced charge transfer and separation, and visible light utilization. The quick recombination rate of the photogenerated narrow excitation wavelength range and charge carriers of photocatalysts, however, limit g-photocatalytic GCN’s activity [55]. However, various ways for improving photocatalytic consistency and performance have been discovered, such as combining GCN with a hole scavenger and a metal cocatalyst to achieve high visible-light photoactivity.

### 4.2. Wastewater and Environmental Treatment

The majority of operations in the petrochemical, petrochemical textile, and food industries contaminate the environment, particularly aquatic bodies. Textiles, photographic materials, and printing materials all use organic dyes, and during the dying process, these colors permeate into the majority of aquatic ecosystems. Although these colors/dyes are harmful to human and environmental health, they are difficult to breakdown biologically and chemically. Because of this danger, an improved oxidation approach for the remediation of contaminated drinking water and nonbiodegradable pollutants is urgently needed. The use of semiconductors such as GCN for photocatalysis has been found in most studies to be the best alternative for wastewater and environmental treatment due to their less harmful nature [56]. The photophysical potentials of the parent nitride can be modified by heteroatom doping, heterojunction formation with other materials, and textural alterations to improve the surface area and porosity, making GCN a potential photocatalyst for the destruction of a range of pollutants. The structure, manufacturing, and fabrication techniques of GCN nanosheets influence the photocatalyst’s efficacy and use in wastewater treatment. To synthesize and develop candidates for water and environmental treatment, several scientists have used doping metals such as Cu and Fe and nonmetals C, B, S, or O, as well as codoping. A remedy to environmental contamination may be found in the combination of noble metals and GCN. To summarize, most of the most well-known photocatalysts’ impractical applications in wastewater and environmental pollution are due to some of their drawbacks, which include high cost, small scale, limited photocatalytic activity, and a form of assumed recycling.

### 4.3. GCN as Photoelectronic Materials

Incorporating nitrogen into carbon improves its performance in terms of electronic, structural, and mechanical properties, mainly its electronic qualities, making it a viable material for use in batteries, light-emitting devices, fuel cells, and solar cells. As a member of the carbon nitride materials, GCN has a bright future in many applications [57]. The sp^2^ hybridization of carbon and nitrogen results in the formation of a π-conjugated electronic structure, which gives outstanding photoelectronic characteristics.

### 4.4. GCN as a Light Emitting Material

**Figure 6:**
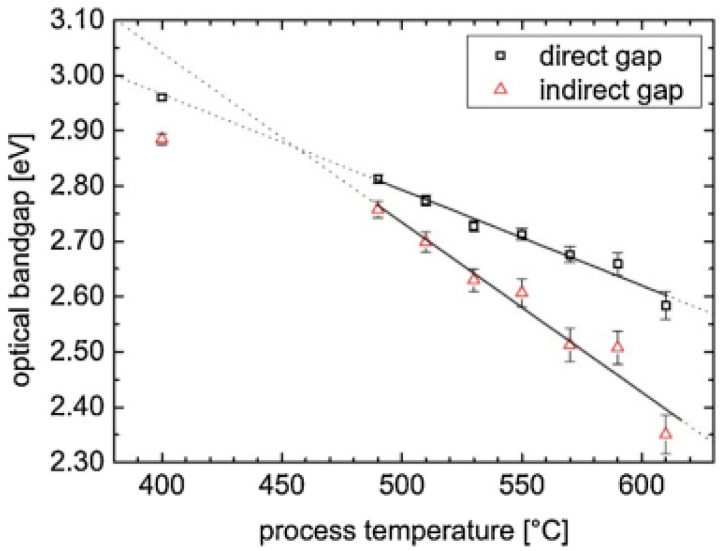
Direct (squares) and indirect (triangles) bandgaps values for carbon nitride powders treated at various temperatures. Linear fits in the range 490 to 610 °C are indicated by solid black lines, whereas extrapolations are indicated by dotted lines. Adapted with permission from [61]. 2014, Elsevier.

Because of its semiconductor features, researchers are currently focusing their efforts on GCN luminescence. The optical bandgap dominates the photoluminescence area in general, and it has been shown that the bandgap of GCN is adjustable with processing temperature [58]. Figure 6 depicts the link between the optical bandgap and synthesized temperature, which is thought to be related to the GCN tectonic unit.

The photoluminescence tunability of GCN was explored in depth by Dong’s group [59,60,61]. The resulting emission region of the samples ranges from 400 to 510 nm, ranging from blue-violet to green, as shown in Figure 6. This novel energy level mechanism diagram of carbon nitride GCN nanopowders is proposed based on the two-dimensional conjugated polymeric network and lone pair of carbon nitride, concluding that the narrowing of bandgap was caused primarily by enhancement of the conjugated polymeric system and electron delocalization.

## 5. Conclusions and Outlook

The benefits, diverse features, design methodologies, and future applications of robust GCN-based composite photocatalysts are highlighted in this review. GCN has been demonstrated as one of the utmost promising possibilities for developing and producing sophisticated composite photocatalysts for a broad spectrum of uses. Although significant progress has been made in recent years, numerous obstacles remain in rationally fabricating highly efficient GCN-based photocatalysts for diverse applications and completely understanding the underlying enhancing process in composite photocatalysis employing GCN. Still, there are many unanswered questions and areas where further study may be done. As a result, additional research is needed to fully exploit the structural and electrical features of GCN composite photocatalysts. Owing to the difficulty in obtaining highly efficient and stable GCN-derived photocatalysts having narrowed bandgaps, the new developments/design of conjugate narrow-band polymers may give alternative concepts for accelerating photocatalysis improvements based on organic semiconductors. Furthermore, precise surface defect control and simple GCN nanosheet scale fabrication procedures are widely sought. Advanced electrocatalysts and photoelectron catalysts based on GCN should be used to expand the applicability of GCN-based photocatalysts in the research community.

The applications of GCN-based photocatalysts have centered mostly on hydrogen generation and pollutant degradation. The CO_2_ photoreduction using GCN-based photocatalysts has grown increasingly appealing during the last three years. The fundamental character of the GCN surface dictates its promising future in CO_2_ reductions. Moreover, the study of O_2_ generation from the opposite half-reaction of water splitting, which involves water splitting as well as CO_2_ reduction, should receive greater attention very shortly. The specific mechanism of the process, notably CO_2_ reduction employing GCN-based photocatalysts, is yet unknown and unsolved. In the future, a thorough understanding of reaction pathways will be essential for exposing the fundamentals of photocatalytic enhancement and designing highly effective GCN-based photocatalysts. Furthermore, some key factors that contribute to elevated photocatalytic activities, such as charge transfer dynamics, optical absorption, and electronic band structure, should be thoroughly investigated using both computational and experimental simulations to gain theoretical insights. From another point of view, GCN-based photocatalysts could give more attention to designing and formulating the more efficient nanostructures that are attentive to morphology tracking, assessing the practicality of photocatalysis. Furthermore, it should also be taken into account in connection with the degrading property and mechanism of other types of contaminants, particularly nondyed pollutant(s), and then investigating the usability of various GCN-based nanoparticles in wastewater treatment, solar energy application, and sensing properties by thoroughly evaluating their photocatalytic potential, expense, energy usage, and reusability. Additionally, future research should primarily focus on using technological advances or combining current approaches to increase the relaxing velocity of GCN to increase the runoff rate, which may be utilized to enhance the material in boosting photocatalytic performance. Sites of reactant adsorption, the kinetics of charge transfer, and molecular orbitals must be thoroughly investigated in terms of experimental effort. Researchers from many areas and nations must work together to elevate GCN-based photocatalysts to a new level of interest.

## Figures and Tables

**Table 1:** Hydrogen production study by differing GCN heterostructure. Adapted with permission from [38]. 2021, Elsevier.

Photocatalytic Material	Light Source	Performance
GCN/Au/CdS	300 W Xenon lamp	530 μmol after 5 h
C,N-TiO_2_/GCN	300 W Xenon arc lamp	39.18 mmol h^−1^ g^−1^
GCN/WS_2_	300 W Xenon arc lamp	101 μmol h^−1^ g^−1^
WO_3_/GCN	Artificial solar light	110 μmol h^−1^ g^−1^
WO_3_/GCN	300 W Xenon	1853 μmol h^−1^ g^−1^
Bi_2_MoO_6_/GCN	300 W Xenon lamp	563.4 μmol h^−1^ g^−1^

**Table 2:** Studies of GCN heterojunctions on carbon dioxide (CO_2_) reduction.

Photocatalytic Material	Light Source	Performance
GCN/ZnO [42]	300 W Xenon arc lamp	0.6 μmol/h·g CH_3_OH
SnO_2_-X/GCN [43]	500 W Xenon lamp	22.7 μmol/h·g CH_3_OH, CO, CH_4_
BiOI/GCN [44]	300 W Xenon arc lamp	17.9 μmol/g CO

## Data Availability

Not applicable.
